# CD8^+^ T cells in breast cancer tumors and draining lymph nodes: PD-1 levels, effector functions and prognostic relevance

**DOI:** 10.1080/2162402X.2025.2502354

**Published:** 2025-05-12

**Authors:** Carolina Abrate, Fernando P. Canale, Sabrina N. Bossio, Jimena Tosello Boari, María C. Ramello, Nicolas Nuñez, Wilfrid Richer, Christine Sedlik, Jordan Denizeau, Anne Vincent-Salomon, Edith Borcoman, Andres Del Castillo, Adriana Gruppi, Eva V. Acosta Rodríguez, Eliane Piaggio, Carolina L. Montes

**Affiliations:** a Departamento de Bioquímica Clínica, Facultad de Ciencias Químicas, Universidad Nacional de Córdoba, Córdoba, Argentina; b Centro de Investigaciones en Bioquímica Clínica e Inmunología (CIBICI-CONICET), Córdoba, Argentina; c Inflammation Research Lab, Institute of Experimental Immunology, University of Zürich, Zürich, Switzerland; d INSERM U932 Immunity and Cancer, Department of Translational Research, PSL Research University, Institut Curie Research Center, Paris, France; e Diagnostic and Theranostic Medicine Division, Institut Curie, PSL Research University, Paris, France; f Department of Medical Oncology, Institut Curie, Paris, France; g Departamento de Mastología y Ginecología – Hospital Rawson, Polo Hospitalario, Córdoba, Argentina

**Keywords:** Breast cancer, CD8^+^ T cells, exhaustion, PD-1, draining lymph nodes

## Abstract

CD8^+^ T cells shape the antitumor immune response. Here, we evaluated CD8^+^ T cells expressing different levels of PD-1, their functional status, and distribution in different tissues of luminal breast cancer (BC) patients. We characterized the exhaustion stages of CD8^+^ T cells in tumors, juxtatumoral tissues (JTs), and tumor-draining lymph nodes (TDLNs). Terminal exhausted CD8^+^ T cells (PD-1^High^ CD8^+^) were predominant in tumors and nearly absent in other tissues. However, in all tissues evaluated, most CD8^+^ T cells exhibited a pre-exhausted phenotype (PD-1^Int^ CD8^+^) or did not express PD-1. PD-1^High^ and PD-1^Int^ CD8^+^ T cells from tumors and JTs presented central and effector memory phenotypes, while in TDLNs were primarily central memory. TCR-β sequencing revealed higher clonality among CD8^+^ T cells from tumor than TDLNs, with tumor-enriched clones also detected in TDLNs. Analysis of scRNA-seq datasets from tumors and JTs of colorectal and non-small cell lung cancer patients, identified a CD8^+^ terminal exhaustion and a CD8^+^ pre-exhausted signatures. High expression of exhaustion-associated genes in BC tumors correlated with improved overall survival. Overall, PD-1 expression effectively distinguishes exhaustion stages in CD8^+^ T cells. PD-1^Int^ cells found in tumors, JTs, and TDLNs represent a promising therapeutic target for cancer immunotherapy.

## Introduction

Tumor-infiltrating CD8^+^ T cells have been associated with favorable clinical outcomes, in solid tumors including melanoma,^1^ lung,^2^ breast cancer (BC),^3^ among others. Antigen recognition by naïve CD8^+^ T cells triggers their clonal expansion, effector differentiation, and the subsequent formation of heterogeneous memory populations: long-lived central memory (CM) T cells and short-lived effector memory (EM) T cells. However, in the tumor microenvironment, persistent antigenic stimulation can impair memory T cell differentiation, leading to a state of T cell exhaustion. Interestingly, exhausted T cell subsets often display memory-like phenotypes, including characteristics of CM and EM cells.^4^

Exhausted CD8^+^ T cells exhibit high expression of inhibitory receptors (IRc), altered transcriptome, epigenome and metabolism, as well as impaired effector function.^5^ Notably, we have described that CD39 expression identifies human and mouse exhausted tumor-infiltrating CD8^+^ T cells.^6^ Emerging evidences highlight that exhausted CD8^+^ T cells display heterogeneous phenotype.^7^ Factors such as tumor histology, persistent antigen stimulation, and antigen-MHC class I binding affinity contribute to varying levels of exhaustion.^5,8^ Pre-exhausted and terminal exhausted CD8^+^ T cells can be identified by differential expression of IRc, transcription factors, effector capacity, and proliferation.^7^ Importantly, this heterogeneity has significant implications for cancer immunotherapy, as the composition of these subsets may influence responses to immune checkpoint blockade.^9^ For instance, pre-exhausted CD8^+^ T cells are capable of robust expansion following anti-PD-L1 therapy, whereas terminally exhausted cells typically remain unresponsive.^10^

Checkpoint blockade antibodies can modulate T-cell function not only within tumors but also during T-cell priming in tumor-draining lymph nodes (TDLNs).^11^ Understanding the immune characteristics of the CD8^+^ T cells targeted by anti-PD-1 therapies is critical for optimizing treatment strategies. However, most studies on T cell exhaustion in BC have focused on primary tumors and blood samples,^12^ leaving the immune landscapes of TDLNs and adjacent non-tumoral mammary tissues largely underexplored.^13,14^

In the present study, we aimed to perform a comprehensive analysis of the phenotype and function of exhausted CD8^+^ T cell subsets. We evaluated not only in tumors but also in juxtatumoral (JTs) breast tissues, metastatic and non-metastatic TDLNs, from patients with luminal BC, with the purpose of defining the features of these subsets and providing clues that may impact therapeutic strategies. Our study revealed that terminal exhausted CD8^+^ T cells (PD-1^High^ CD8^+^) were predominantly present in tumors and nearly absent in other tissues. We demonstrated that most CD8^+^ T cells in tumors, JTs, and TDLNs from BC patients exhibited a pre-exhausted phenotype (PD-1^Int^ CD8^+^) or did not express PD-1. Using phenotypic profiling approaches, we showed that assessing PD-1 expression levels is an effective approach for identifying different subsets of exhausted CD8^+^ T cells across various tissues of BC patients. Moreover, we defined a gene signature including CD8A, PDCD1, TOX, and cytotoxic molecules in tumors, which correlated with better survival in BC patients.

## Materials and methods

### Human samples

Tumor samples (invasive), adjacent non-tumoral breast tissue (JT), metastatic (*M*-) and non-metastatic (NM-) TDLNs, were collected from 81 untreated female BC patients undergoing standard surgical resection at Institut Curie Hospital (Paris, France) and Hospital Rawson (Córdoba, Argentina). Sentinel lymph nodes were excluded due to institutional restrictions on research use. JT was defined as follows: for small specimens, a 1 cm distance from the margin was maintained, whereas for larger specimens, the margin distance ranged from 8 to 10 cm.

All patients were free of chronic infections, and had not receive any treatment prior to surgery. Clinical information of the participants is provided in Supplementary Table S1. The hormone receptor status of the BC cohort included in our study: ER+ (13%), HER2+ (3%), ER+PR+ (82%), HER2+ER+ (1%).

The study was conducted following the ethical principles outlined in the Declaration of Helsinki. Ethical approval was obtained from the Institutional Review Board and Ethics Committee of the Institut Curie Hospital Group and the Polo Hospitalario Provincia de Córdoba. Written informed consent form was obtained from all participants. Histological analysis classified TDLNs as either M-DLNs or NM-DLNs based on the presence of tumor cells, confirmed by EpCAM/CD45 staining using flow cytometry. Tumors and TDLNs were disaggregated mechanically and enzymatically with Colagenasa D (0.5 U/mL) and DNase I (50 U/mL) (Roche).

### Flow cytometry

Cell suspensions from tumors, TDLNs, and JTs were labeled with monoclonal antibodies targeting human antigens (Supplementary Table S2). Gating strategies used to identify cellular populations of different tissues from BC patients are depicted in Supplementary Figure S1a,b. Frequency of Tumor-Infiltrating CD8^+^ T cells in BC patients with different hormone receptor status is shown in Supplementary Figure S1c.

Transcription factor (intracellular staining) and intracellular cytokine determination were performed following protocols previously described and routinely used by our group.^6,15,16^

For cytotoxic molecules determination, cell suspensions were stimulated with phorbol 12-myristate 13-acetate (PMA) (Sigma) at 100 ng/ml and Ionomycin (Sigma) at 2 ug/ml in the presence of Brefeldin A (eBioscience), Monensin (eBioscience) and CD107a antibody for 5 h at 37 °C. After stimulation, cells were fixed and permeabilized with fixation/permeabilization solution (eBiosciences), according to the manufacturer’s instructions.

Samples were acquired in a BD LSR Fortessa flow cytometer (BD Biosciences) and the data analyzed with FlowJO^TM^ software. To obtain an unbiased overview, we systematically reduced the flow cytometry data to two dimensions by applying Uniform Manifold Approximation and Projection (UMAP) algorithm,^17^ which displayed randomly selected 16,000 live leukocytes from TDLNs, JTs and tumors of 4 patient samples. Clusters were annotated according to CD3, CD8, CD4, B220 and FOXP3 expression.

### Cell sorting for TCR sequencing

CD8^+^ T cells from matched NM-DLNs, M-DLNs and tumors from 3 BC patients were isolated using Pan T isolation kit as previously described.^13^ Memory CD8^+^ T cells (CD8+CD27-/+CD45RA-/+) were sorted by flow cytometry in a BD FACS ARIA II cell sorter (purity of 98–99%). Gating strategy in Supplementary Figure S1d.

### TCR-sequencing methodology

The reverse transcription (RT) reaction was carried out using primers targeting the constant TCRβ region, which were attached at the 5’ end to the common sequence 2 (CS2: TACGGTAGCAGAGACTTGGTCT, using SuperScript IV. Then, cDNA was purified with the Agencourt RNAclean XP kit (Beckman Coulter) following the manufacturer’s protocol (Ambion).

The cDNA amplification and barcoding involved three PCR steps. In the first PCR, the same reverse primers used in the RT were used along with previously published forward TCR primers.^18^ The conditions: 17 cycles of 95°C for 3 minutes, 90°C for 30 seconds, 63°C for 30 seconds, and 72°C for 30 seconds. Then, cDNA purification was performed using the Agencourt AMPure XP kit.

The second PCR step involved two seminested multiplex PCRs for TCRβ, employing variable region primers^18^ that were appended with the common sequence 1 (CS1: ACACTGACGACATGGTTCTACA). The first PCR product served as the template. Amplification was performed using GoTaq G2 hot Start polymerase (Promega), under the conditions: 30 cycles of 95°C for 3 minutes, 90°C for 30 seconds, 63°C for 30 seconds, and 72°C for 30 seconds. After amplification, cDNA purification was performed with the Agencourt AMPure XP kit.

For the final PCR, barcoding and pair-end sequencing were added using the PE1_CS1 forward primer and PE2_barcode_CS2 reverse primer (Fluidigm). This step used the second PCR product as the template, with Platinium Taq DNA Polymerase High Fidelity (ThermoFisher). The PCR conditions: 94°C for 10 minutes, followed by 30 cycles of 94°C for 30 seconds, 60°C for 30 seconds, and 68°C for 4 minutes, with a final extension at 68°C for 3 minutes. The amplified cDNA was purified as previously described. Each PCR product was barcoded and included a Fluidigm paired-end sequence, enabling sequencing on an Illumina MiSeq system.

### CS2-TRBC primer sequence

The common sequence (CS2) used in TCR amplification is as follows:

**TACGGTAGCAGAGACTTGGTCTT**ACCAGTGTGGCCTTTTGGGTGTG

The common sequence is indicated in bold.

### TCR-seq analysis

TCR sequencing data were analyzed using MiXCR^19^ with default parameters to extract and quantify CDR3 sequences. TCRβ clones were identified based on the V, J, and CDR3 sequences extracted from the MiXCR output. To minimize noise, we filtered out clones with fewer than three counts and selected the top 90% most abundantly expressed TCRβ clones for each sample. TCR-seq data has been deposited in Gene Expression Omnibus database with the GEO accession number GSE289478.

### TCGA data, survival analysis and scRNA-seq data

Gene expression data and survival information for BC patients were obtained from TCGA platform through the cBioPortal website (https://www.cbioportal.org/datasets). The analysis was carried out using data from 817 patients with invasive BC. The analysis was based on mRNA expression results that were previously normalized and scaled using the z-score method. First, patients with high PTPRC (CD45) expression were filtered using the median expression as a cutoff point, considering all available samples. The process was then repeated by selecting patients with high CD8A expression. TOX expression was subsequently categorized as high or low by defining the cutoff point that maximized the log-rank statistic of a Kaplan-Meier model for overall survival, using the “surv cutpoint” function from the R package “survminer”. Once patients defined as PTPRC^High^, CD8A^High^, TOX^High^ were identified, the expression of the genes PDCD1, PRF1 and GZMB was re-categorized using the “surv cutpoint” function. Survival probability plots were generated using the “survfit” and “ggsurvplot” functions as instructed by the TCGA data package.

The scRNA-seq datasets were obtained from publicly available repositories. Processed data from Non-Small Cell Lung Cancer (NSCLC) and Colorectal Cancer (CRC) were obtained from Gene Expression Omnibus database (GEO) (accession numbers GSE99254 and GSE108989).^20^ CD8^+^ T cell clusters are as originally annotated through unsupervised clustering methods. Comparisons were made between the distribution of the clusters in different tissues as well as the expression of specific genes between cell subsets.

### Statistical analysis

GraphPad Prism 9.0.0 software was used for statistical analysis. Samples from two different experimental groups were considered unpaired, whereas samples involving cell populations obtained from the same individual were considered paired. The statistical test used for each comparison is detailed in each figure and was chosen according to the sofware’s recommendations. *p* values ≤ 0.05 were considered statistically significant.

## Results

### CD8^+^ T cells present in different tissues exhibit distinct states of differentiation and clonal expansion

**Figure 1.**
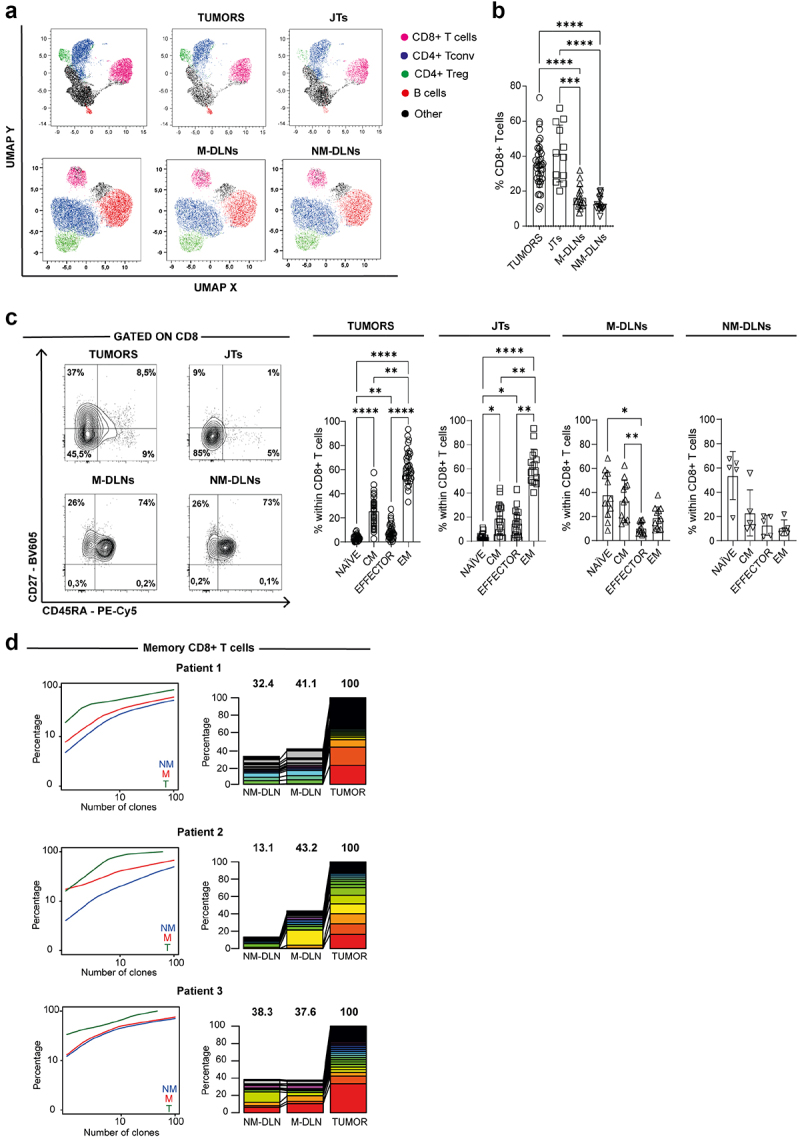
CD8^+^ T cells from tumors, JTs and *M*- and NM-DLNs from BC patients. (a) UMAP projection showing the clustering of 16,000 leukocytes in tumors, JTs, M-DLNs and NM-DLNs from BC patients (N = 4). Each color represents a cluster and is associated with a different immune population defined by phenotypic markers (CD3, CD8, CD4, CD19, FOXP3). (b) Frequencies of CD8^+^ T cells within CD45^+^ cell population in tumors (circle, N = 39), JTs (square, N = 12), M-DLNs (triangle, N = 17) and NM-DLNs (inverted triangle N = 19). (c) Representative contour plots (left panel) and frequencies (right panel) of cells expressing CD45RA and/or CD27 within CD8^+^ T cells in tumors (circle, N = 34), JTs (square, N = 16), M-DLNs (triangle, N = 12) and NM-DLNs (inverted triangle, N = 5). (d) Next-generation-sequencing-based high-throughput TCR-β CDR3 analysis of sorted memory CD8^+^ T cells from matched NM- and M-DLNs and tumors from 3 BC patients. The cumulative frequencies of TCR-β CDR3 clones in memory CD8^+^ T cells (left panel), from NM-DLN (blue), M-DLN (red) and tumor (green). Overlap of the TCR-β CDR3 clones in memory CD8^+^ T cells (right panel), from NM-DLN, M-DLN and tumor. The top 100 TCR-β CDR3s from tumor memory CD8^+^ T cells were identified, and the percentage of these clones found in both NM- and M-DLN are shown. *p*- values were calculated using Kruskal-Wallis (b) or Friedman test (c). Data presented as mean ± SD, *p ≤ 0.05, **p ≤ 0.01, ***p ≤ 0.001, ****p ≤ 0.0001. Only significant differences are indicated.

Knowledge about the specific infiltrate of immune cells and their functionality in patients with cancer is important for prognosis and therapy design.^21^ We first applied high-dimensional flow cytometry analysis, using the dimensionality reduction algorithm UMAP, to evaluate the distribution of leukocytes (CD45^+^) subsets in tumors, JTs and TDLNs from BC patients. The corresponding analysis led us to calculate the proportion and distribution of CD8^+^ T cells, CD4^+^ Treg cells, conventional CD4^+^ T cells and B cells clusters among the 4 tissues evaluated (Figure 1a). The quantification of the CD8^+^ T cells among CD3^+^ population revealed that, in tumors and JTs the frequencies of CD8^+^ T cells were similar: 35.62 ± 13.50% and 41.45 ± 16.25% respectively. Interestingly, we observed that M-DLNs and NM-DLNs exhibited lower frequencies of CD8^+^ T cells than tumor and JTs (Figure 1b).

Using the CD27 and CD45RA markers, we identified CD8^+^ T cells subsets: naïve (CD45RA^+^, CD27^+^), central memory (CD45RA^−^, CD27^+^), effector memory (CD45RA^−^, CD27^−^), and effector (CD45RA^+^, CD27^−^). We observed that the EM phenotype was the most represented in both tumors and JTs, whereas the naïve CD8^+^ T cell population was the least abundant in these tissues. In M-DLNs, we observed that the frequencies of naïve and CM CD8^+^ T cells were higher compared to the effector CD8^+^ T cells meanwhile in NM-DLNs the naïve CD8^+^ subset was the most represented (Figure 1c). The comparative analysis of CD8^+^ T cells according to their differentiation stages across the evaluated tissues revealed that the tumors and JTs exhibited higher infiltration of CD8^+^ T cells with an EM phenotype compared to M-DLNs and NM-DLNs. Conversely, naïve CD8^+^ T cells were more represented in M-DLNs and NM-DLNs compared to tumor and JTs (Supplementary Figure S2a). These data suggest as preferential accumulation of activated T cells both in tumor and JTs.s

Given that memory T cells are characterized as cells that have encountered their specific antigen and are present in both tumors and TDLNs, we next investigated the diversity of the TCR repertoire within the memory CD8^+^ T cell subset from matched M-DLNs, NM-DLNs, and tumors from BC patients.

To assess T cell repertoire clonality, we created cumulative frequency plots of TCR-β CDR3 sequences derived from memory CD8^+^ T cells across three tissue types (Figure 1d). We found that the TCR repertoire was less diverse in tumors compared to TDLNs. Approximately 20,000 tumor-infiltrating CD8^+^ T cells were predominantly represented by a limited number of clones, with 220, 61, and 47 dominant clones identified in patients 1, 2, and 3, respectively (Supplementary Table S3). To further explore the overlap of TCR repertoires across tissues, we identified the top 100 most expanded CDR3 sequences in the tumor, as these likely represent key antitumor clones. Then, their distribution was examined in both M-DLNs and NM-DLNs. Interestingly, a significant proportion of these tumor-expanded CD8^+^ T cell clones were also detected in both M-DLNs and NM-DLNs. These results suggest that a substantial fraction of the most expanded CD8^+^ T cell clones present in the tumor are also present in TDLNs, underscoring their potential importance in antitumor immunity.

### CD8^+^ T cell subsets with differing in PD-1 expression levels and differentiation statuses are unevenly distributed across tumor, juxtatumoral tissue, and draining lymph nodes

**Figure 2.**
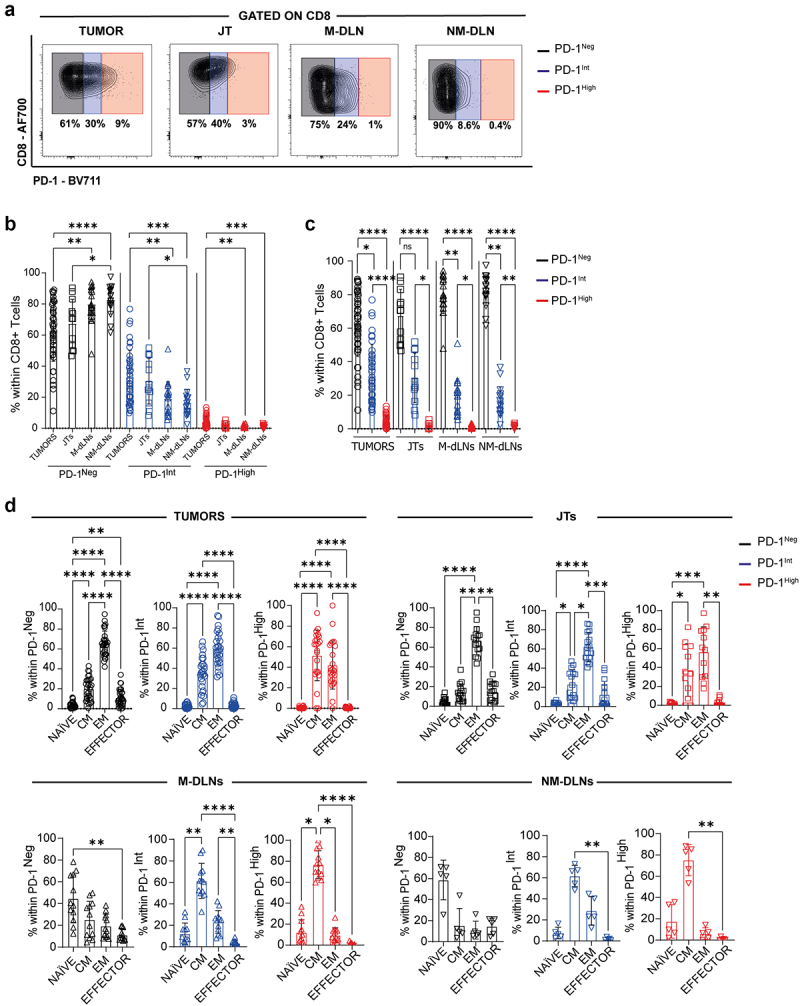
PD-1 expression on CD8^+^ T cells from different tissues in BC patients. (a) Representative contour plots of PD-1 expression on CD8^+^ T cells from tumors, JTs, M-DLNs and NM-DLNs from BC patients. (b) Frequencies of PD-1^Neg^ (black), PD-1^Int^ (blue) or PD-1^High^ (red) CD8^+^ T cells in tumors (circle, N = 37), JTs (square, N = 12), M-DLNs (triangle, N = 19) and NM-DLNs (inverted triangle, N = 19) – comparison between tissues. (c) Comparison of frequencies of PD-1^Neg^ (black), PD-1^Int^ (blue) or PD-1^High^ (red) CD8^+^ T within tumors (circle, N = 37), JTs (square, N = 12), M-DLNs (triangle, N = 19) and NM-DLNs (inverted triangle, N = 19) (d) Frequency of NAÏVE, central memory (CM), EFFECTOR or effector memory (EM) CD8^+^ T cells within PD-1^Neg^ (black), PD-1^Int^ (blue) or PD-1^High^ (red) CD8^+^ T cells in tumors (circle, N = 20), JTs (square, N = 15), M-DLNs (triangle, N = 12) and NM-DLNs (inverted triangle, N = 5). p-values were calculated using Kruskal-Wallis (b) or Friedman test (c). Data presented as mean ± SD, *p ≤ 0.05, **p ≤ 0.01, ***p ≤ 0.001, ****p ≤ 0.0001. Only significant differences are indicated.

The different stages of exhaustion and the functionality of CD8^+^ T cells in BC patients have not been fully elucidated. While the expression of PD-1 represents a hallmark of CD8^+^ T cell exhaustion, recent reports reveal that varying degrees of PD-1 expression enable the identification of different exhausted CD8^+^ T cell subsets.^22^ For instance, high PD-1 expression correlates with a terminal exhaustion stage, while intermediate PD-1 expression corresponds to a pre-exhaustion stage.^7^ To identify the heterogeneity within the exhausted CD8^+^ T cell population in tumors and different tissues of BC patients, CD8^+^ T cells were classified as PD-1^High^ or PD-1^Int^ and compared to PD-1^Neg^ (Figure 2a). Our analysis showed that tumors exhibited higher frequency of PD-1^High^ and PD-1^Int^ CD8^+^ T cells compared to TDLNs (M and NM) (Figure 2b). PD-1^High^ CD8^+^ T cells constitute a minor population present almost exclusively in tumors, with an even minor representation in JTs. PD-1^Int^ and PD-1^Neg^ CD8^+^ T cell populations remained the predominant subsets in tumors, JTs and TDLNs (Figure 2c).

Furthermore, our multi-parametric flow cytometry analysis enabled us to evaluate the differentiation status of these PD-1-expressing CD8^+^ T cell subsets (Supplementary Figure S3a,b). As depicted in Figure 2d, tumor-infiltrating PD-1^High^ and PD-1^Int^ CD8^+^ T cells showed CM and EM phenotypes, whereas PD-1^Neg^ CD8^+^ T cell subsets were predominantly represented by the EM phenotype. Similarly, PD-1^High^ CD8^+^ T cells from JTs displayed the same phenotype distribution as those from tumors, whereas PD-1^Int^ and PD-1^Neg^ CD8^+^ T cells were predominantly EM. In contrast, in M-DLNs and NM-DLNs, PD-1^High^ and PD-1^Int^ subsets exhibited a CM phenotype, with a less prominent EM phenotype (Figure 2d). These results, in line with a previous report^23^ highlights that PD-1-expressing CD8^+^ T cells represent an activated population. Notably, we detected that the PD-1^Neg^ CD8^+^ T lymphocytes identified in tumors and JTs also exhibited an EM phenotype, whereas in TDLNs, it mainly corresponded to non-activated or naïve T cells (Figure 2d).

### PD-1^High^ CD8^+^ T cells express markers of terminal T cell exhaustion

**Figure 3.**
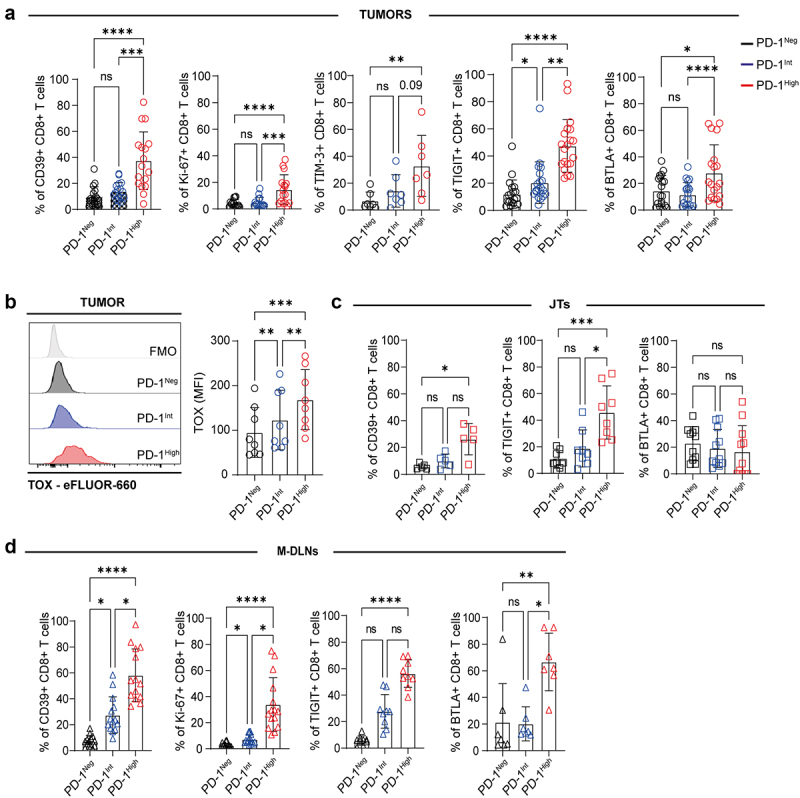
Expression of exhaustion markers on CD8^+^ T cells from different tissues in BC patients. (a) Frequency of CD39^+^, Ki-67^+^, TIM-3^+^, TIGIT^+^ or BTLA^+^ within PD-1^Neg^ (black), PD-1^Int^ (blue) and PD-1^High^ (red) tumor-infiltrating CD8^+^ T cells. (b) Representative histograms of TOX expression within tumor-infiltrating CD8^+^ T cells. Fluorescence minus one (FMO) (gray), PD-1^Neg^ (black), PD-1^Int^ (blue), PD-1^High^ (red) (left panel). MFI of TOX in PD-1^Neg^ (black) PD-1^Int^ (blue) and PD-1^High^ (red) tumor-infiltrating CD8^+^ T cells (right panel). (c) Frequencies of CD39^+^, TIGIT^+^, or BTLA^+^ within PD-1^Neg^ (black), PD-1^Int^ (blue) and PD-1^High^ (red) CD8^+^ T cells in JTs samples. (d) Frequencies of CD39^+^, Ki-67^+^, TIGIT^+^, or BTLA^+^ within PD-1^Neg^ (black), PD-1^Int^ (blue) and PD-1^High^ (red) CD8^+^ T cells in M-DLNs samples. p-values were calculated using Friedman test (a) or RM one-way ANOVA Tukey test using Geisser-Greenhouse correction (b). Data presented as mean ± SD, *p ≤ 0.05, **p ≤ 0.01, ***p ≤ 0.001, ****p ≤ 0.0001, ns: non-significant differences.

Subsequently, we examined the expression of exhaustion-associated molecules on CD8^+^ T cells displaying varying levels of PD-1 in tumors, JTs, and M-DLNs. As depicted in Figure 3a and Supplementary Figure S4a, we detected a higher percentage of tumor-infiltrating PD-1^High^ CD8^+^ T cells expressing CD39, Ki-67 and IRc (TIGIT and BTLA) compared to PD-1^Int^ and PD-1^Neg^ CD8^+^ T cell populations. Interestingly, we observed that tumor-infiltrating PD-1^High^ CD8^+^ T cells showed higher expression of the transcription factor TOX than PD-1^Int^ and PD-1^Neg^ CD8^+^ T cells populations (Figure 3b), further confirming their exhausted phenotype. In JTs, we found that PD-1^High^ CD8^+^ T cells showed higher frequencies of CD39 and TIGIT expressing cells compared to PD-1^Neg^ CD8^+^ T cells (Figure 3c and Supplementary Figure S4b). In M-DLNs, all CD8^+^ T subsets defined by PD-1 expression levels exhibited a similar expression profile to tumor-infiltrating CD8^+^ T cell subsets. Thus, PD-1^High^ CD8^+^ T cells exhibited higher expression of CD39, Ki67, TIGIT, and BTLA compared to PD-1^Neg^ CD8^+^ T cell population (Figure 3d and Supplementary Figure S4c). Additionally, we found a positive correlation between PD-1 expression and the exhaustion markers described above in tumor-infiltrating CD8^+^ T cells, suggesting that PD-1 expression levels are associated with the exhaustion stage of CD8^+^ T cells within BC tumors (Supplementary Figure S5a-c).

### PD-1^Int^ and PD-1^High^ CD8^+^ T cells from tumors and metastatic draining lymph nodes exhibits differential cytokine production and cytotoxic potential

**Figure 4.**
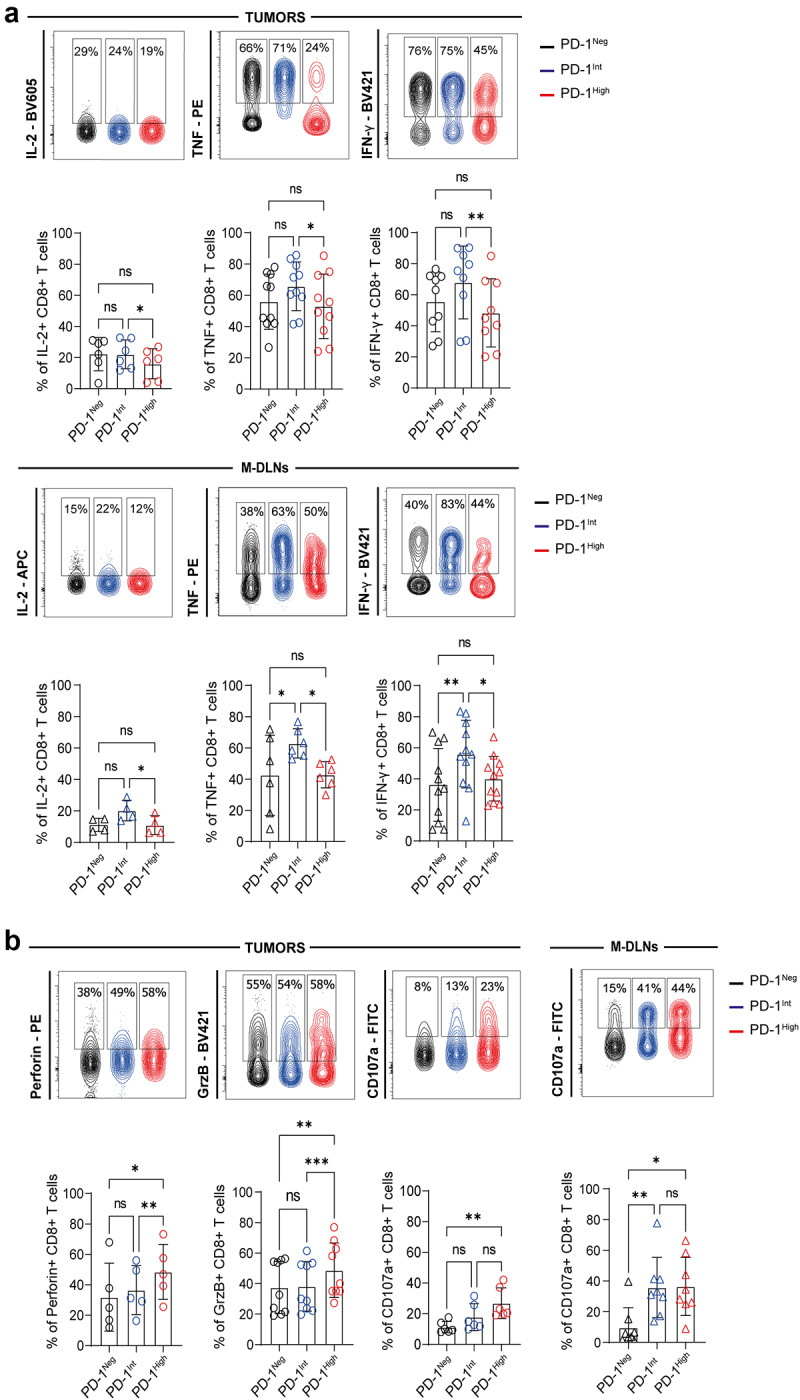
Effector function of CD8^+^ T cells from tumors and M-DLNs in BC patients. (a) Representative contour plots and frequencies of cytokine-producing CD8^+^ T cells (IL-2, TNF and IFN-γ) within PD-1^Neg^ (black), PD-1^Int^ (blue) and PD-1^High^ (red) in tumors (top panel) and M-DLNs (bottom panel) after PMA/Ionomycin stimulation. (b) Representative contour plots and frequencies of Perforin^+^, GrzB^+^ and CD107a^+^, within PD-1^Neg^ (black), PD-1^Int^ (blue) and PD-1^High^ (red) tumor-infiltrating CD8^+^ T cells after PMA/Ionomycin stimulation (left panel) and CD107a^+^ within PD-1^Neg^ (black), PD-1^Int^ (blue) and PD-1^High^ (red) CD8^+^ T cells in M-DLNs (right panel). p-values were calculated using Friedman test. Data presented as mean ± SD, *p ≤ 0.05, **p ≤ 0.01, ***p ≤ 0.001, ns: non-significant differences.

Previous reports have demonstrated that exhausted CD8^+^ T cells exhibit a reduced capability to produce effector cytokines depending on the stage of T cell exhaustion.^7^ Moreover, terminally exhausted CD8^+^ T cells have been found to display elevated expression of cytotoxic molecules.^24^ To evaluate the effector function of CD8^+^ T cells with different expression levels of PD-1, we analyzed cytokine production and the expression of cytotoxicity-associated molecules in CD8^+^ T cells from tumors and M-DLNs following PMA/Ionomycin stimulation. PD-1^High^ CD8^+^ T cells exhibited lower frequencies of IL-2, TNF and IFNγ-producing cells, than PD-1^Int^ CD8^+^ T cells both in tumors and M-DLNs (Figure 4a). We found that within tumors, PD-1^High^ CD8^+^ T cells displayed the highest frequencies of Perforin, Granzyme B (GrzB) and CD107a expressing cells following *ex vivo* stimulation. However, in M-DLNs, we detected that both PD-1-expressing subsets showed higher CD107a expression than the PD-1^Neg^ ones (Figure 4b).

### High gene expression of PD-1 and cytotoxicity-associated molecules in the tumor microenvironment (TME) correlates with increased survival in breast cancer patients

**Figure 5.**
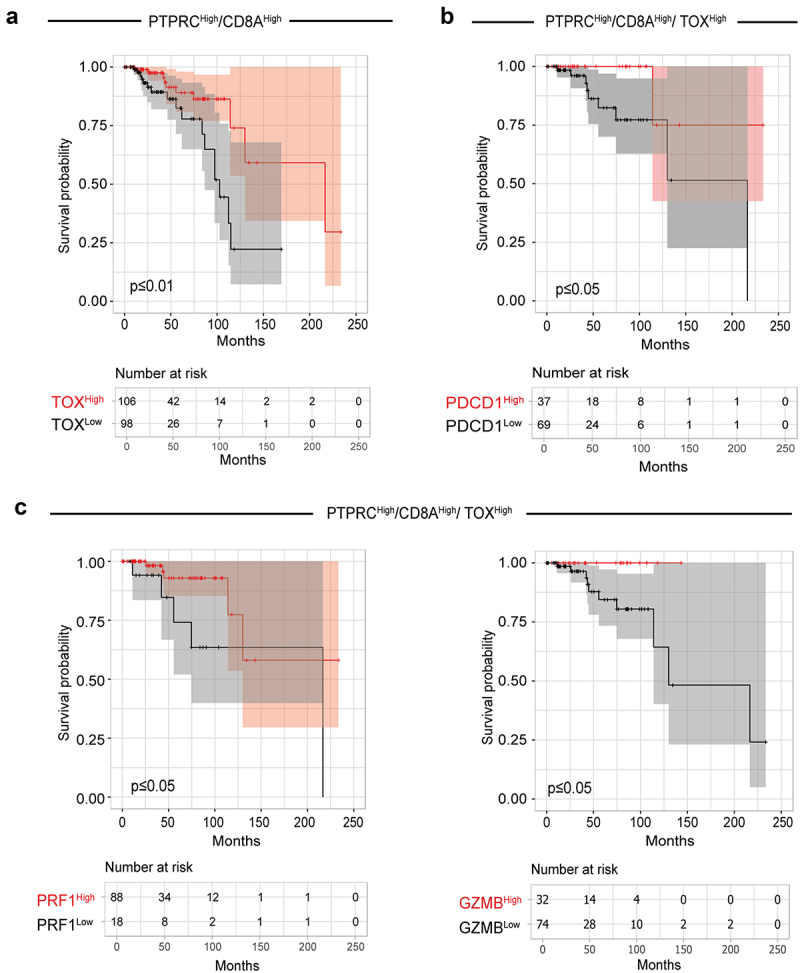
High expression of exhaustion markers correlates with better overall survival in BC patients. (a) Kaplan-Meier curves comparing the survival probability of BC patients (TCGA cohort) with high expression of PTPRC (CD45) and CD8A genes, stratified by expression levels of TOX. (b) Kaplan-Meier curves comparing the survival probability of the BC patient’s cohort with high expression of PTPRC (CD45), CD8A and TOX genes stratified by expression levels of PDCD1 (PD-1) or, (c) PRF1 (perforin) and GZMB (GrzB). The number of patients in each selected population is indicated in the graph. The survival curves show the 95% confidence interval of the analysis shaded in the plot. The p-value for the survival analysis shown in the graph is derived from the log-rank test.

Our results revealed that tumors from BC patients are infiltrated by PD-1^Int^ and PD-1^High^ CD8^+^ T cells that exhibit traits of pre and terminal exhaustion respectively. Given the current limitation of lacking a definitive gene signature to distinguish pre-exhausted CD8^+^ T cells, we sought to investigate the potential impact of exhaustion-associated factors including the gene expression of TOX, PD-1, and cytotoxic molecules (Perforin and GrzB) on the clinical prognosis of patients with invasive breast carcinoma. We analyzed overall survival in a cohort of patients from the TCGA consortium, based on the gene expression of these markers derived from bulk RNA sequencing of tumor tissues. We initially classified samples based on high expression of the PTPRC (CD45) and CD8A genes, to select cases with significant CD8^+^ T cells-infiltration. Subsequently, the probability of survival in the defined patient cohort was calculated distinguishing between patients with high and low expression of TOX. We observed that high TOX expression in the TME correlates with a higher probability of survival for BC patients (Figure 5a). Next, we evaluated the contribution of PDCD1 (PD-1) expression in those TMEs with high expression of PTPRC, CD8A, and TOX. We found that patients with high PDCD1 expression in their TME had a better probability of survival compared to those with low expression of this gene (Figure 5b).

Finally, within tumors with high expression of PTPRC, CD8A, and TOX, we evaluated the contribution of the expression of genes associated with cytotoxicity such as PRF1 (Perforin), and GZMB (GrzB). The analyses indicated that patients exhibiting high expression of cytotoxicity-associated genes had a higher probability of survival compared to patients with low expression of these genes (Figure 5c). These results suggest that, in BC patients with high immune infiltration, a gene expression signature characterized by high levels of CD8A, PDCD1, TOX and cytotoxic molecules is associated with improved overall survival.

### scRNA-seq analysis identifies CD8-LAYN and CD8-GZMK clusters with gene signatures of terminal and pre- T cell exhaustion

**Figure 6.**
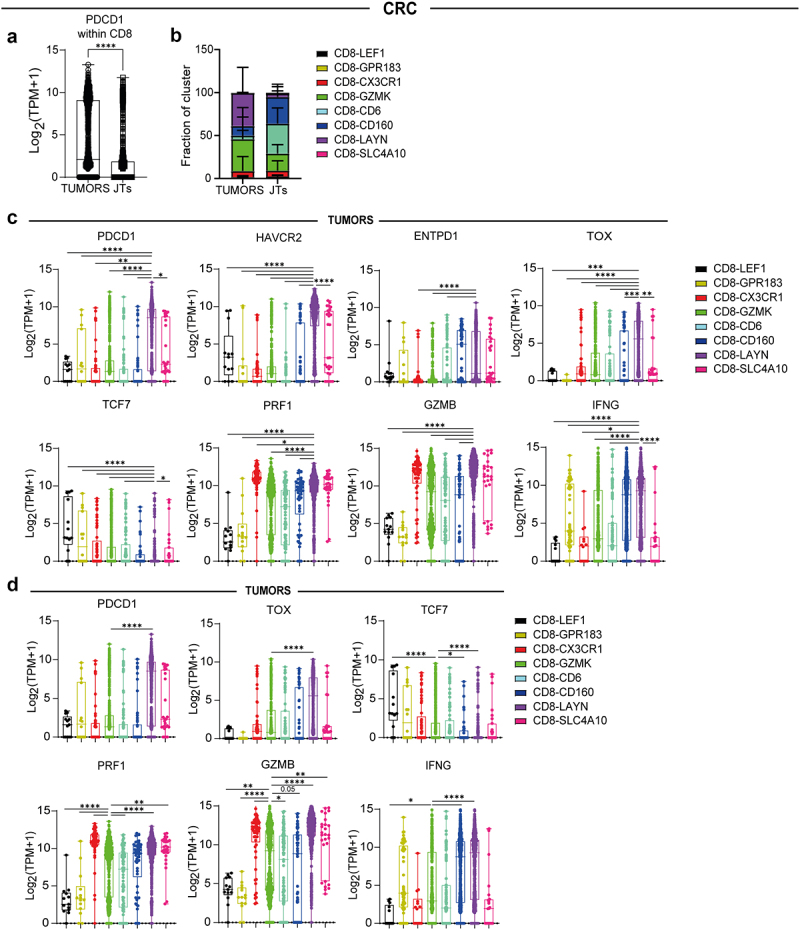
scRNA-seq data reveal CD8^+^ T cell clusters exhibiting pre-exhausted and terminally exhausted signatures in tumors and JTs from CRC patients. (a) PDCD1 (PD-1) gene expression in CD8^+^ T cells from tumors and JTs. (b) Distribution of eight clusters of CD8^+^ T cells across tumors and JTs. (c) Analysis of PDCD1 (PD-1), HAVCR2 (TIM-3), ENTPD1 (CD39), TOX, TCF7, PRF1 (perforin), GZMB (GrzB) and IFNG (IFN-γ) gene expression on CD8^+^ T cells clusters of single cells isolated from tumors. Comparison between CD8-LAYN (term-ex) and other clusters. (d) Analysis of PDCD1 (PD-1), TOX, TCF7, PRF1(Perforin), GZMB (GrzB) and IFNG (IFN-γ) gene expression on CD8^+^ T cells clusters of single cells isolated from tumors. Comparison between CD8-GZMK (pre-ex) and other clusters. Clusters were defined in the publication^20^. p-values were calculated using Dunnett’s multiple comparisons test. Data presented as mean ± SD, *p ≤ 0.05, **p ≤ 0.01, ***p ≤ 0.001 and ****p ≤ 0.0001. Only significant differences are indicated.

Next we aim to identify CD8^+^ T cells at different exhaustion stages in more immunogenic tumor microenvironments. We analyzed publicly available scRNA-seq datasets of CD8+ T cells from tumors and JTs from patients with CRC^20^ and NSCLC.^25^ We observed significantly higher PDCD1 gene expression in CD8^+^ T cells from tumors compared to JTs in both CRC and NSCLC patients (Figure 6a and Supplementary Figure S6a).

In their original work, Zhang et al.^20^ identified eight distinct CD8^+^ T cell clusters in both tumors and JTs of CRC patients. To explore cluster distribution patterns, we compared the frequency of these CD8^+^ T cell clusters between tumor and JT samples. Notably, CD8-LAYN and CD8-GZMK clusters, were the predominant in tumors. Moreover, CD8-LAYN was highly enriched in tumors and nearly absent in JTs (*p* < 0.0001), while CD8-GZMK was present in both tumor and JT samples (Figure 6b).

Upon further analysis we found that CD8-LAYN cluster exhibited the highest expression levels of terminal exhaustion markers, including PDCD1, HAVCR2, ENTPD1, TOX, PRF1, GZMB, and IFNG, along with the lowest TCF7 expression compared to other CD8^+^ T cell clusters (Figure 6c). In contrast, the CD8-GZMK cluster displayed a gene expression profile indicative of pre-exhausted CD8^+^ T cells, characterized by higher TCF7 gene expression and lower levels of PDCD1, TOX, and cytotoxicity-related genes (PRF1, GZMB, and IFNG) than the CD8-LAYN cluster (Figure 6d). Similar results were observed in an NSCLC dataset (Supplementary Figure S6c-d).

## Discussion

Tumor-infiltrating CD8^+^ T cells have been in the spotlight of tumor immunology. Our study is the first to provide a detailed comparative analysis of CD8^+^ T cells across multiple tissue compartments, including tumors, JTs, M-DLNs and NM-DLNs in BC patients. This broader perspective offers a more complete understanding of the immune landscape in BC. Our work demonstrates that not only tumors but also JTs in patients with BC are infiltrated by CD8^+^ T cells in remarkably similar frequencies. Moreover, our data suggests that most of those infiltrating CD8^+^ T cells have an EM phenotype, suggesting they are antigen-experienced T cells. Our results are consistent with previous studies that show elevated expression of inflammatory genes in breast adenocarcinomas compared to resected healthy breast tissue obtained during mammoplasty reduction surgery, and support the notion that adjacent tumoral tissue is a valuable source of information about the TME in which the tumor develops.^26^ In line with our data, Chen et al.^27^ reported that in NSCLC, adjacent non-neoplastic samples exhibited higher infiltration of CD8^+^ T, naïve B, Myeloid and NK cells than tumor samples, suggesting that the two regions could provide complementary prognostic value in early-stage NSCLC. Interestingly it has been reported that CD103^+^ Tissue Resident Memory (TRM) cells are a major component of CD8^+^ TILs in human triple negative BC patients (TNBC) and nearly all CD8^+^ T cells in noncancerous breast tissue express CD103.^28^

We are the first to demonstrated that Memory CD8^+^ T cells from the tumor exhibited a higher clonality than those in TDLNs. RNAseq analysis of the β-CDR3s showed that high frequencies of tumor-enriched T cell clones were present in the TDLNs suggesting that tumor-specific T cells traffic between primary tumor and the TDLNs. Moreover, these results underscore the contribution of TDLNs as a source of tumor-specific T cell targets that might be modulated by immunotherapies.

In this work, we observed that the major difference between infiltrating CD8^+^ T cells in tumor and the JTs, M-DLNs and NM-DLNs was the presence of PD-1^High^ -expressing cells. In fact, these PD-1^High^ CD8^+^ T cells are nearly absent in the other tissues. Instead, PD-1^Int^ and PD-1^Neg^ CD8^+^ T cell populations remain the predominant subsets in all tissues analyzed. Besides, we found a positive correlation between PD-1 expression and the frequency of tumor-infiltrating CD8^+^ T cells expressing exhaustion markers such as CD39, IRc, cytotoxic molecules and/or TOX, suggesting that tumor-infiltrating PD-1^High^ CD8^+^ T cells in BC tumors exhibit a terminal exhaustion phenotype. Our data is consistent with previous reports that revealed that exhausted CD8^+^ T cells in chronic infection and cancer are a heterogenous population.^10,29^ Progenitor exhausted CD8^+^ T cells can be identified by intermediate expression of PD-1 and expression of the chemokine receptor CXCR5, while terminally exhausted cells display high PD-1 levels along with Tim-3 and other IRc.^30,31^ Transcriptomic analysis of these tumor-infiltrating CD8^+^ T cells subsets in murine B16 and human melanoma tumors reveal that progenitor exhausted cells are enriched in *Slamf6* and lack of ENTPD1 *(CD39)* and HAVCR2 *(Tim-3)* expression.^32^ Additionally, exhaustion markers like HAVCR2 and ENTPD1 are significantly upregulated in PD-1^High^ compared to PD-1^Int^ and PD-1^Neg^ T cells.^32^

Our results are novel in demonstrating that while pre-exhausted CD8^+^ T cells are present in tumors, JTs and TDLNs, the terminally exhausted T cell population is almost exclusively found in tumors from BC patients. Interestingly, it has been shown that distinct subsets of exhausted CD8^+^ T cells exhibit differences in their anatomical location. For example, Beltra et al.^7^ demonstrated that progenitors exhausted CD8^+^ T cells are found in circulation and peripheral organs, meanwhile terminal exhausted T cells are present in inflamed tissues, such as tumors. In concordance, the existence of higher frequencies of PD-1 expressing CD8^+^ T cells in TDLNs compared with non-TDLNs has been reported in several solid mouse tumor models, irrespective of cancer type, mouse genetic background or tumor localization.^33^

We observed that CD8^+^ T cells from tumors and JTs, regardless of PD-1 expression, exhibit a differentiation stage corresponding to CM and EM. Furthermore, we did not find naïve CD8^+^ T cells in these tissues. In TDLNs, CD8^+^ T cells with high and intermediate PD-1 expression displayed an EM and CM phenotype, and as expected, naïve T cells correspond to PD-1^Neg^ T cells. Likewise, Egelstone et al.^28^ demonstrated that tumor-infiltrating CD8^+^ T cells from melanoma and BC patients were both primarily composed of EM cells. In other tumor scenarios, it has been reported a significant increase of EM CD8^+^ T cells in ascitic fluid compared to both the peripheral blood of healthy individuals and ovarian cancer patients. Conversely, the frequencies of naïve and effector CD8^+^ T cells are reduced in these ascitic samples compared to peripheral blood.^34^ Together, these data clearly point to the preferential accumulation of antigen experienced EM cells in the tumor tissue. We speculate that tumor-infiltrating PD-1^Neg^ CD8^+^ T cells represent an earlier phase of effector differentiation compared to PD-1 expressing tumor-infiltrating CD8^+^ T cells and would therefore be predicted to have effector potential and capacity to sustain long-lasting immunity.

Our results demonstrate that, compared to PD-1^Int^ cells, tumor-infiltrating PD-1^High^ CD8^+^ T cells exhibit reduced cytokine production but increased cytotoxic capacity (perforin, GrzB) and CD107 expression. This observation aligns with previous report,^24,35^ which shows that terminally exhausted CD8^+^ T cells retain enhanced cytotoxic potential despite impaired cytokine secretion. Similarly, Guo et al.^12^ reported that tumor-infiltrating CD8^+^ T cells with high PD-1 expression exhibit lower (but not absent) percentages of IL-2 and TNF-producing cells compared to PD-1^lo^ CD8^+^ T cells.

Together, these findings support the idea that exhausted CD8^+^ T cells are not fully dysfunctional, but rather they represent a heterogeneous population, with subsets retaining effector functions that may contribute to tumor control.

Checkpoint blockade immunotherapy is associated with improved CD8^+^ T cell-mediated anti-tumor responses. PD-1/PD-L1 blocking antibodies are believed to act primarily in TME by re-invigorating pre-exhausted CD8^+^ T lymphocytes and reviving the immune response against tumor.^36^ Hence, the distribution of tumor-infiltrating CD8^+^ T cells with varying stages of exhaustion may have implications for the design of therapeutic strategies as well as for prognosis evaluation.

Interestingly, in patients with NSCLC that respond to anti-PD-1 therapy but not in non-responders, increased number of pre-exhausted T cells were observed.^37^ It has been suggested that pre-exhausted T cells do not arise from the reinvigoration of terminally exhausted cells; rather, they accumulate through local expansion and replenishment by peripheral T cells with both new and pre-existing clonotypes.^37^

More recently, the dogma that PD-1/PD-L1-blockade occurs primarily at the tumor site has been challenged. In fact, Dammeijer et al.^33^ demonstrated that targeting PD-L1 in both TDLNs and periphery induces enhanced anti-tumor CD8^+^ T cell immunity by seeding the tumor site with pre-exhausted T cells. This finding demonstrates that T cells from TDLNs are capable of generating checkpoint blockade-mediated immunity. Interestingly, checkpoint blockade therapy also induces an expansion of antigen-specific PD-1^Neg^ tumor-infiltrating CD8^+^ T cells, which can contribute to the therapeutic effect.^38^ Thus, the combination of Tim-3 and PD-1 blockade resulted in changes in the transcriptional profile of PD-1^Neg^ tumor-infiltrating CD8^+^ T cells. Of note, the PD-1^Neg^ CD8^+^ T cell subset included antigen-specific cells with effector and memory-precursor-like phenotype, with effector and proliferative capacity.^38^ In addition, it has been demonstrated that checkpoint blockade can act on different immune cells within the TME to stimulate anti-tumor CD8^+^ T cell responses.^39,40^

PD-1/PD-L1 and CTLA-4 blockade have given impressive clinical results in various tumor types.^41^ In TNBC, one of the most encouraging immunotherapies is the anti-PD-1/PD-L1 monotherapy. Given that checkpoint-blocking antibodies can exert their effects not only during the effector phase of T cells within the TME, but also during the priming phase of T cells in the TDLNs, it is crucial to understand the immune status of CD8^+^ T cells that can be targeted by immunotherapy. Our work is pioneering in demonstrating the presence of CD8^+^ T cells with different levels of exhaustion and high frequencies of tumor-enriched T cell clones in the TDLNs of BC patients. We believe that our data provide the rational basis for re-evaluating surgical procedures involving the removal of the entire lymph node chain during surgeries in cancer patients. While tumor cells’ presence in sentinel lymph node is a negative prognostic factor in many tumor types, the therapeutic benefit of immediate removal of the remaining TDLNs remains controversial. In cervical cancer,^42^ prophylactic DLNs removal improves overall survival; however, in BC patients, it has no effect on outcomes.^43^

Our work demonstrates that tumors from BC patients are infiltrated with a population of PD-1^High^ CD8^+^ T cells with increased TOX expression and cytotoxic potential. The survival analysis in BC patients revealed that an expression signature including high CD8A, PDCD1, TOX and cytotoxic molecules is associated with improved overall survival. Cytotoxic CD8^+^ T lymphocytes have been shown to be associated with better survival of colorectal,^44^ lung,^45^ epithelial ovarian,^46^ renal cell^47^ cancers among others. Moreover, a strong intra-tumoral infiltrate of cytotoxic CD8^+^ T lymphocytes was found to be significantly associated with improved overall survival in BC, independent of standard prognostic and predictive factors such as age at diagnosis, histological grade, ER, PR and HER-2 status, among others.^48^ In addition, Alcaraz-Sanabria et al.^49^ demonstrated that a gene signature including IFNG, CXCL13, and PRF1 correlate with better overall survival and with a higher level of tumor immune infiltrates (B cells, CD4^+^ and CD8^+^ T cells, DCs) in basal-like BC. Tumors of BC patients with high expression of any of the checkpoint molecules, PD-1, LAG-3, CTLA-4 and TIM-3 exhibit better survival following systemic treatments compared to those with a low expression level of the same molecule.^50^

While PD-1 expression in CD8^+^ T cells has been documented in TNBC patients,^12^ our study expands this understanding by demonstrating that PD-1 expression is a reliable marker of exhaustion across other BC subtypes, including ER+, PR+ and HER-2+. This finding enhances the clinical relevance of PD-1 as a biomarker for identifying exhausted T cell subsets across different BC subtypes.

In addition, we found that regardless of the cancer type, tumors are infiltrated by pre-exhausted and terminally exhausted CD8^+^ T cells. Furthermore, we suggest that PD-1 expression level serves as a marker for identifying CD8^+^ T cells with varying degrees of exhaustion, which could have important implications for personalized treatment strategies.

Elucidating the role and the expression levels of PD-1 on CD8^+^ T cells in the TME and TDLNs could provide critical insights for designing new strategies to enhance the effectiveness of existing immunotherapies and also offer a clearer understanding of their potential as predictive markers for therapy outcomes.

## Supplementary Material

Supplemental Material

## Data Availability

The data supporting the article’s findings is available within the article and its supplementary materials. TCR-seq data has been deposited in Gene Expression Omnibus database with the GEO accession number in process. Further data can be provided upon reasonable request to the corresponding author: cmontes@unc.edu.ar

## Abbreviations

BC: breast cancer
TDLNs: tumor draining lymph nodes
M-DLNs: metastatic draining lymph nodes
NM-DLNs: non-metastatic draining lymph nodes
IRc: inhibitory receptors
OS: overall survival
pre-ex: pre-exhausted
GrzB: Granzyme B
TME: tumor microenvironment
