# Supplementary material for: CD8+ T cells in breast cancer tumors and draining lymph nodes: PD-1 levels, effector functions and prognostic relevance
Source: Oncoimmunology. 2025 May 12;14(1):2502354. doi: 10.1080/2162402X.2025.2502354 (PMC12077459; doi:10.1080/2162402X.2025.2502354)
Supplement: Supplemental Material [file KONI_A_2502354_SM7390.zip › New folder/Supplementary Table 1.docx]

**Clinical and Pathological data of recruited/analyzed Breast Cancer Patients**

| \| **N° OF PATIENTS** \| 81 \| \| --- \| --- \| \|  \|  \| \| **TYPE OF SAMPLE** \|  \| \| Tumor \| 59 \| \| Juxtatumor \| 20 \| \| M-DLNs \| 36 \| \| NM-DLNs \| 22 \| \|  \|  \| \| **AGE (years)** \| 31-83 \| \|  \|  \| \| **HISTOLOGY** \|  \| \| Invasive ductal carcinoma (IDC) \| 39 \| \| Invasive lobular carcinoma (ILC) \| 10 \| \| Others \| 24 \| \| Information not available \| 8 \| \|  \|  \| \| **STAGE** \|  \| \| I \| 3 \| \| II \| 34 \| \| III \| 33 \| \| IV \| 1 \| \| Information not available \| 10 \| \|  \|  \| \| **HORMONE RECEPTOR STATUS** \|  \| \| Estrogen receptor (ER) + \| 66 \| \| Progesterone receptor (PR) + \| 57 \| \| HER2+ \| 4 \| \| TN \| 3 \| |
| --- | --- | --- | --- | --- | --- | --- | --- | --- | --- | --- | --- | --- | --- | --- | --- | --- | --- | --- | --- | --- | --- | --- | --- | --- | --- | --- | --- | --- | --- | --- | --- | --- | --- | --- | --- | --- | --- | --- | --- | --- | --- | --- | --- | --- | --- | --- | --- | --- | --- | --- | --- | --- | --- | --- | --- | --- |

**Supplementary Table 1**
