# Supplementary material for: CD8+ T cells in breast cancer tumors and draining lymph nodes: PD-1 levels, effector functions and prognostic relevance
Source: Oncoimmunology. 2025 May 12;14(1):2502354. doi: 10.1080/2162402X.2025.2502354 (PMC12077459; doi:10.1080/2162402X.2025.2502354)
Supplement: Supplemental Material [file KONI_A_2502354_SM7390.zip › New folder/Supplementary Table 2.docx]

**Kits and Clone antibodies and fluorochromes**

| **Antibodies** | | | |
| --- | --- | --- | --- |
| **ANTIGEN** | **FLUOROCHROME** | **CLONE** | **BRAND** |
| CD45RA | PE-Cy5 | HI100 | BD |
| CD27 | BV605 | O323 | Biolegend |
| CD3 | BV650 | SK7 | Biolegend |
| CD45 | APC Cy7 | 2D1 | BD |
| CD4 | BV785 | OKT4 | Ozyme |
| FOXP3 | PE | 236A/E7 | eBioscience |
| CD19 | Alexa 700 | HIB19 | BD |
| CD8 | AF700 | 3B5 | Life Technologies |
| CD39 | PerCP-Cy5.5 | A1 | Biolegend |
| PD-1 | BV711 | EH12.2H7 | Biolegend |
| TIM-3 | FITC | F38-2E2 | Biolegend |
| TIGIT | PerCP eF710 | MBSA43 | eBioscience |
| Ki-67 | PE-Cy7 | Ki-67 | MQ1-17H12 |
| BTLA | PECF594 | J168-540 | BD |
| TOX | eFluor-660 | TXRX10 | Invitrogen |
| TNF | PE | MAb11 | eBioscience |
| IFN-y | BV421 | B27 | BD |
| IL-2 | APC | MQ1-17H12 | BD |
|  | BV605 | MQ1-17H12 | MQ1-17H12 |
| CD107a | FITC | eBioH4A3 | eBioscience |
| PERFORINA | PE | B-D48 | Biolegend |
| GRANZIMA B | BV421 | GB11 | BD |
| **STANING AND PURIFICATION KITS** | | | |
| **KIT NAME** | **CODE** | **BRAND** | |
| LIVE/DEAD Fixable Violet Dead Cell Stain Kit |  | Invitrogen | |
| 4',6-diamidino-2-phenylindole (DAPI) LIVE/DEAD stain |  |  | |
| Pan T Cell isolation kit | 130-096-535 | Miltenyi Biotec, | |

**Supplementary Table 2**
