# Supplementary material for: CD8+ T cells in breast cancer tumors and draining lymph nodes: PD-1 levels, effector functions and prognostic relevance
Source: Oncoimmunology. 2025 May 12;14(1):2502354. doi: 10.1080/2162402X.2025.2502354 (PMC12077459; doi:10.1080/2162402X.2025.2502354)
Supplement: Supplemental Material [file KONI_A_2502354_SM7390.zip › New folder/Supplementary Table 3.docx]

**TCR repertoires in memory CD8^+^ T cells**

| **Patient** | **Sample type** | **Number of sorted memory CD8+ cells** | **TCR beta** | | |
| --- | --- | --- | --- | --- | --- |
|  |  |  | **Total number of reads** | **Number of clones** | **% of shared clones*** |
| 1 | NM-DLN | 108234 | 105195 | 2044 | 32.4 |
|  | M-DLN | 104987 | 138580 | 1608 | 41.1 |
|  | TUMOR | 17979 | 46523 | 220 | 100.0 |
| 2 | NM-DLN | 56300 | 331221 | 1583 | 13.1 |
|  | M-DLN | 55161 | 105246 | 970 | 43.2 |
|  | TUMOR | 19563 | 37236 | 61 | 100.0 |
| 3 | NM-DLN | 100842 | 112890 | 992 | 38.3 |
|  | M-DLN | 64363 | 117987 | 561 | 37.6 |
|  | TUMOR | 3683 | 67167 | 47 | 100.0 |

* among top 100 clones in the tumor

**Supplementary Table 3**
