# Supplementary figures and images for: CD8+ T cells in breast cancer tumors and draining lymph nodes: PD-1 levels, effector functions and prognostic relevance
Source: Oncoimmunology. 2025 May 12;14(1):2502354. doi: 10.1080/2162402X.2025.2502354 (PMC12077459; doi:10.1080/2162402X.2025.2502354)

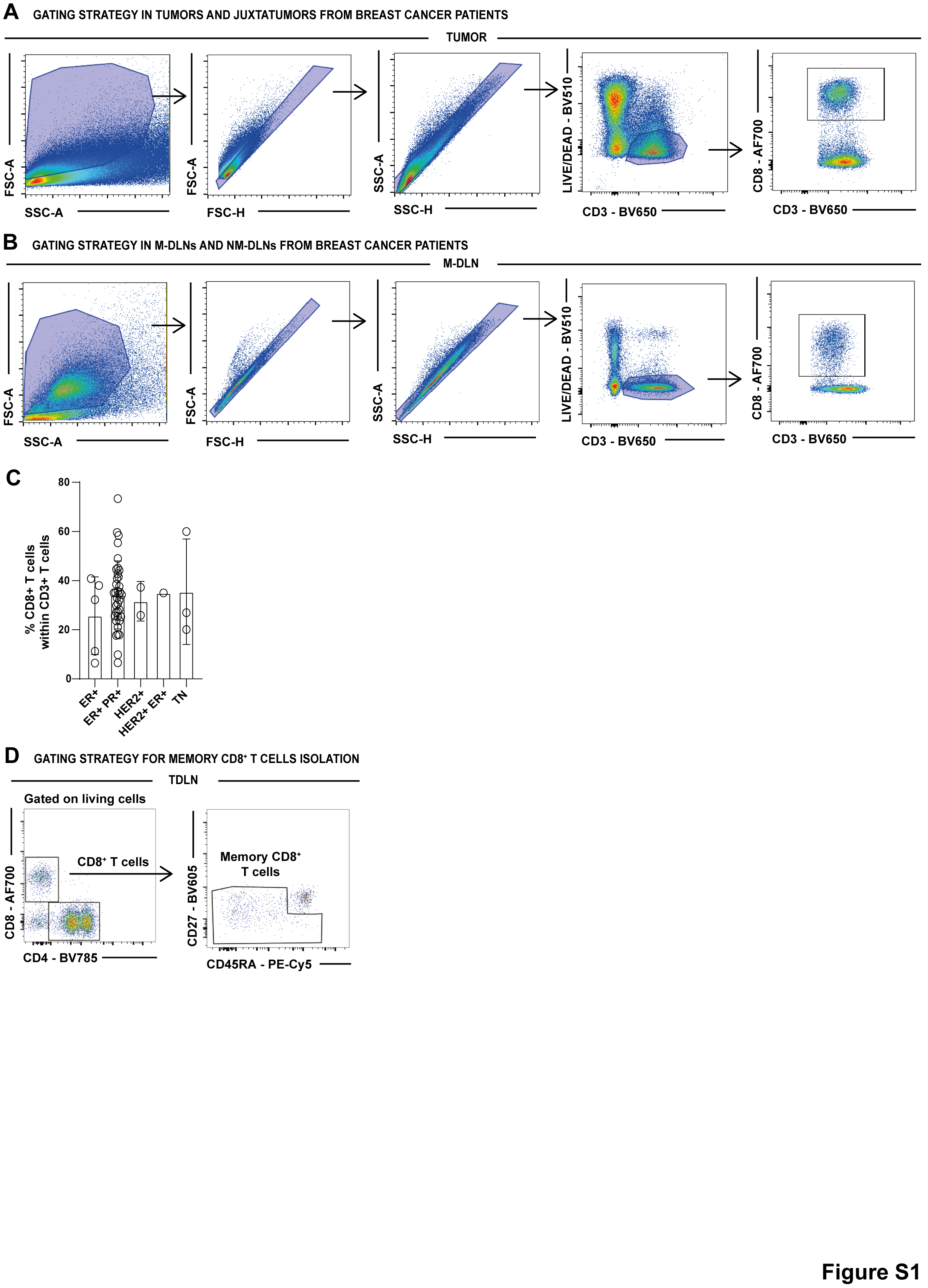

Supplement: Supplemental Material [file KONI_A_2502354_SM7390.zip › New folder/Figure S1 modified.tif]

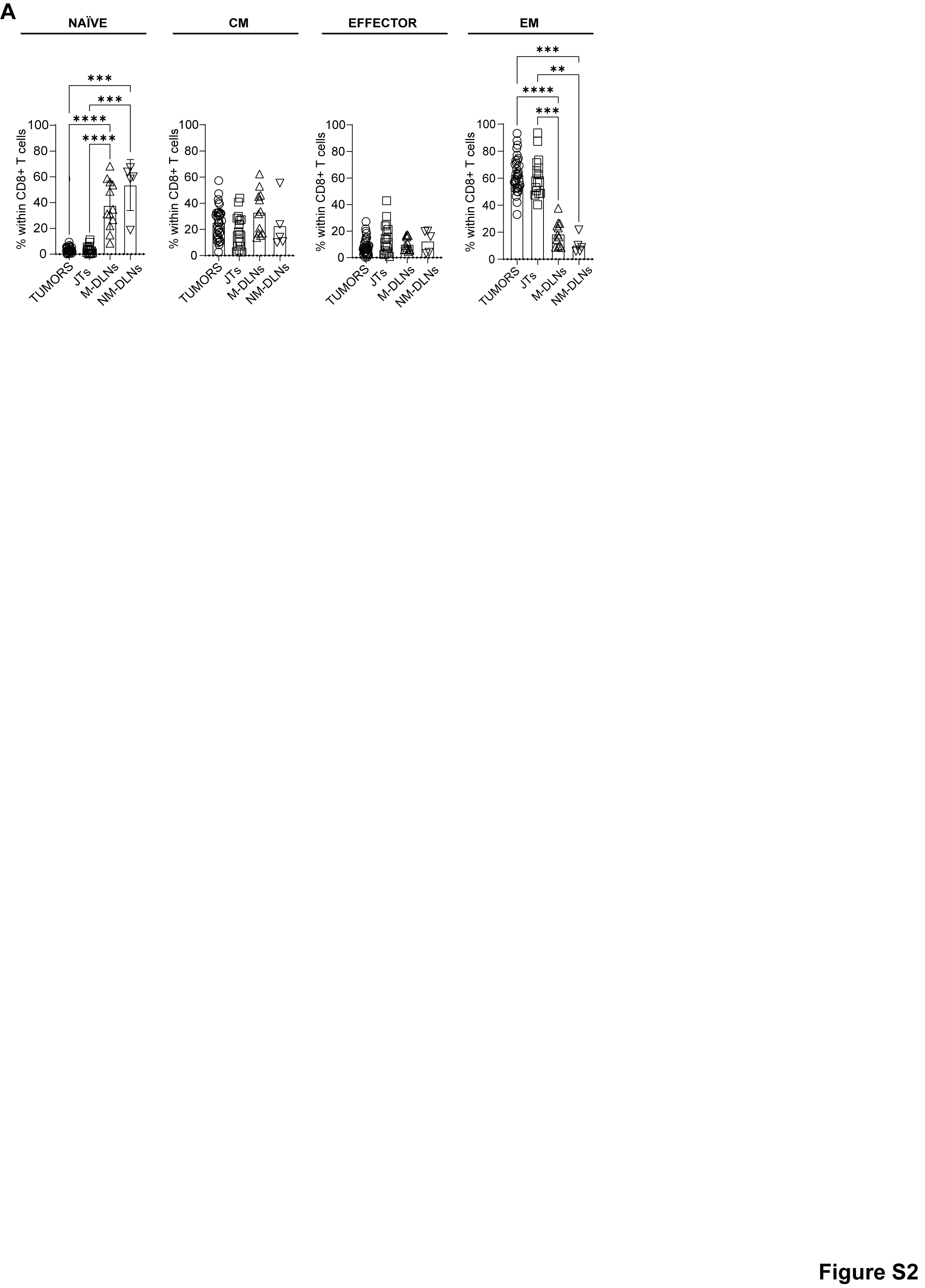

Supplement: Supplemental Material [file KONI_A_2502354_SM7390.zip › New folder/Figure S2.tif]

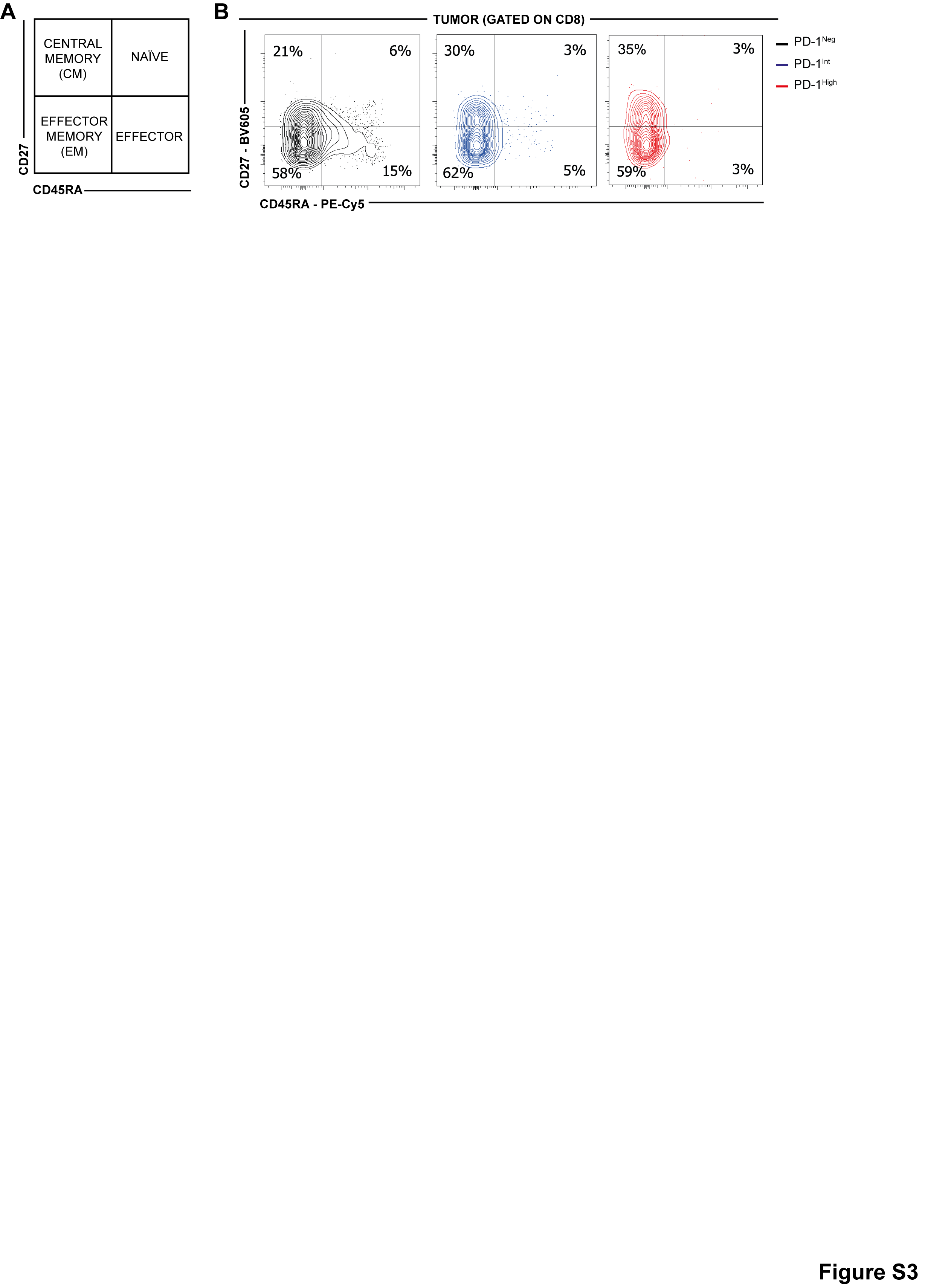

Supplement: Supplemental Material [file KONI_A_2502354_SM7390.zip › New folder/Figure S3.tif]

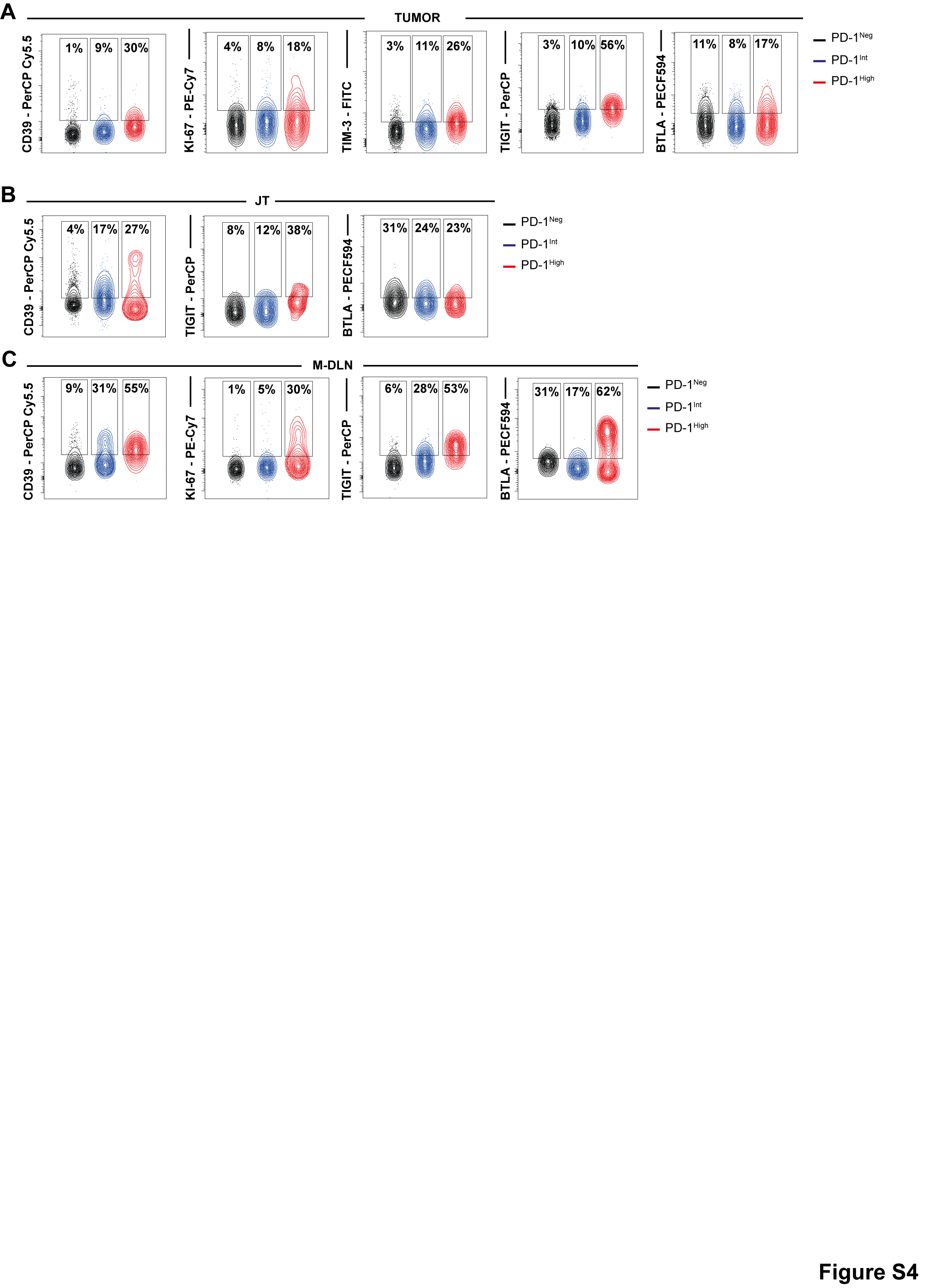

Supplement: Supplemental Material [file KONI_A_2502354_SM7390.zip › New folder/Figure S4.tif]

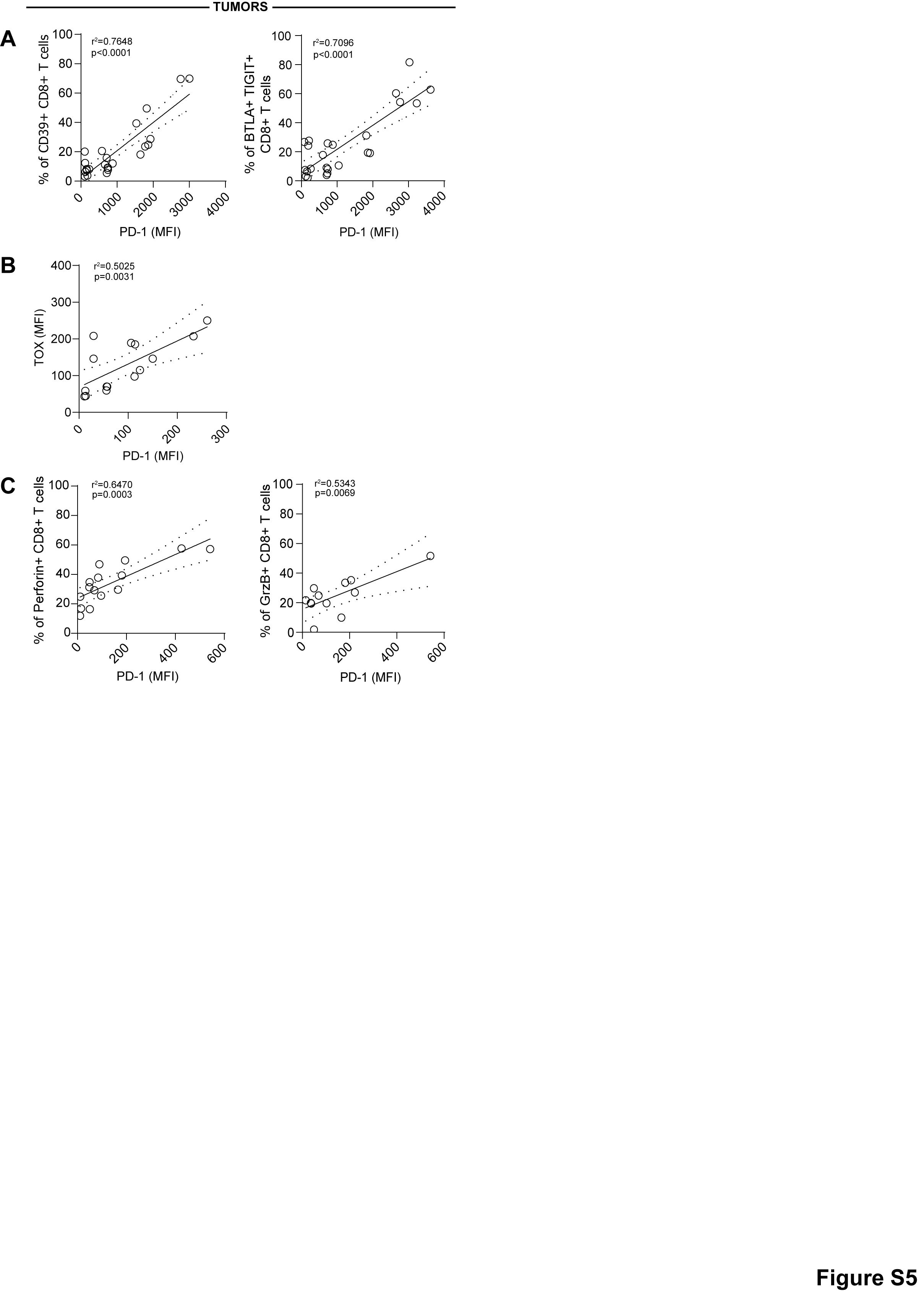

Supplement: Supplemental Material [file KONI_A_2502354_SM7390.zip › New folder/Figure S5.tif]

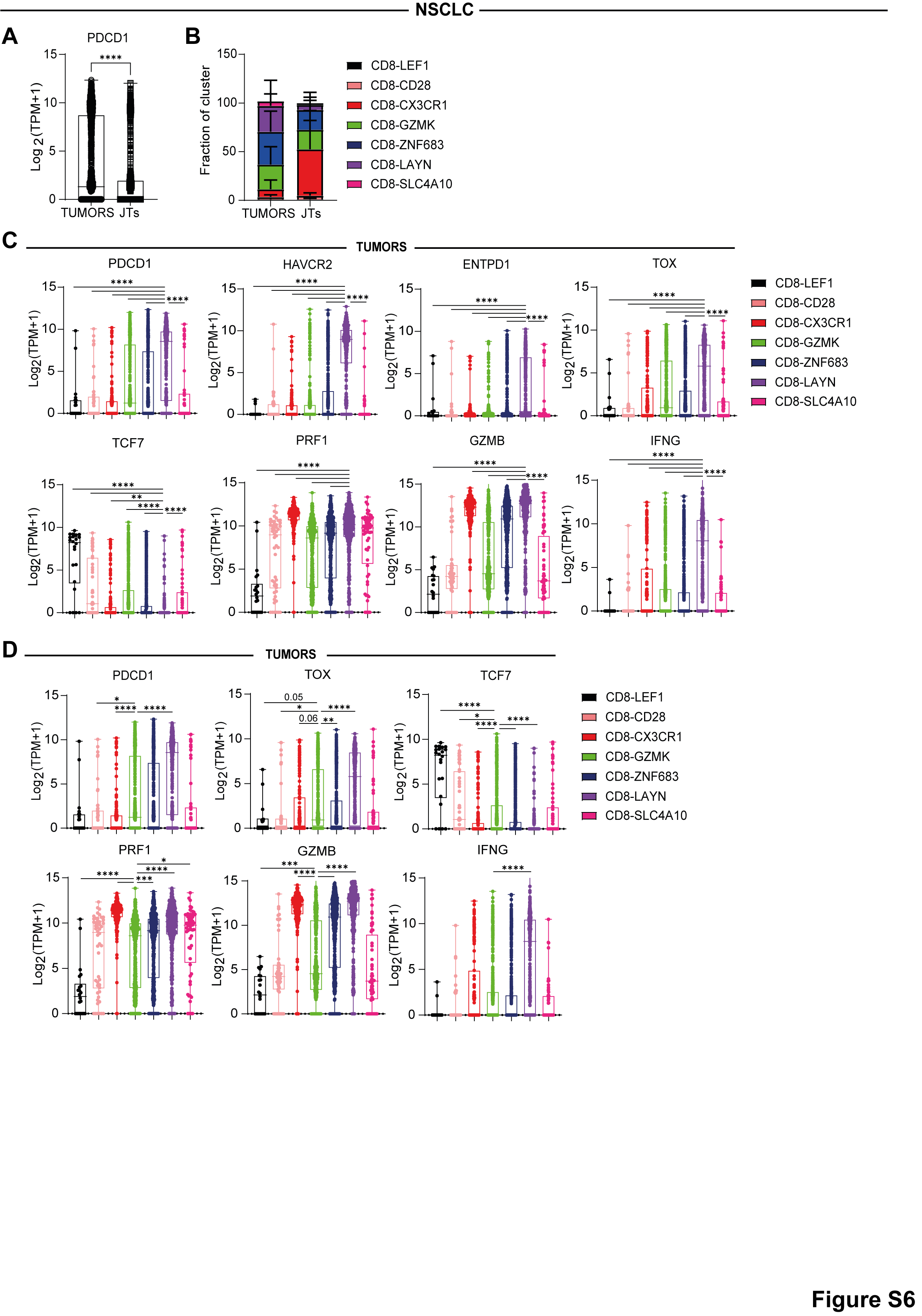

Supplement: Supplemental Material [file KONI_A_2502354_SM7390.zip › New folder/Figure S6.tif]
